# Glycolytic enzyme PKM2 regulates cell senescence but not inflammation in the process of osteoarthritis: PKM2 regulates chondrocyte senescence via p16 ^INK4a^ transcription

**DOI:** 10.3724/abbs.2023062

**Published:** 2023-07-31

**Authors:** Bo Liu, Chenzhong Wang, Ziyu Weng, Yi Yang, Hong Zhao, Yueqi Zhang, Qinming Fei, Yi Shi, Chi Zhang

**Affiliations:** 1 Department of Orthopedic Surgery Zhongshan Hospital Fudan University Shanghai 200032 China; 2 Biomedical Research Centre Zhongshan Hospital Fudan University Shanghai 200032 China

**Keywords:** chondrocyte, PKM2, p16 ^INK4a^, senescence, metabolic reprogramming

## Abstract

Chondrocyte senescence is an important mechanism underlying osteoarthritis in the senile population and is characterized by reduced expressions of the extracellular matrix proteins. The involvement of glycolysis and the tricarboxylic acid cycle in the development of osteoarthritis is inclusive. The present study aims to investigate the role of the glycolytic enzyme M2 isoform of pyruvate kinase (PKM2) in chondrocytes in senescence and inflammation. Primary chondrocytes are isolated from the knee joints of neonatal mice. Small interfering RNAs (siRNAs) against PKM2 are transfected using lipofectamine. RNA sequencing is conducted in primary chondrocytes with the *PKM2* gene deleted. Cell apoptosis, autophagy, reactive oxygen species measurement, and senescent conditions are examined. The glycolytic rate in cells is measured by Seahorse examination. Interleukin 1-β (IL-1β) increases the protein expressions of matrix metallopeptidases (MMP)13 and PKM2 and reduces the protein expression of collagen type II (COL2A1) in primary chondrocytes. Silencing of *PKM2* alters the protein expressions of MMP13, PKM2, and COL2A1 in the same pattern in quiescent and stimulated chondrocytes. RNA sequencing analysis reveals that *PKM2* silencing reduces senescent biomarker p16 ^INK4a^ expression. Compared with low-passage chondrocytes, high-passage chondrocytes exhibit increased expression of p16 ^INK4a^ and reduced expression of COL2A1. Silencing of *PKM2* reduces SA-β-Gal signals and increases COL2A1 expression in high-passage chondrocytes. Seahorse assay reveals that *PKM2* deletion favors the tricarboxylic acid cycle in mitochondria in low- but not in high-passage chondrocytes. In summary, the glycolytic enzyme PMK2 modulates chondrocyte senescence but does not participate in the regulation of inflammation.

## Introduction

Osteoarthritis is a common degenerative joint disease, especially in the senile population, showing joint pain, stiffness, and even disability. Pathological examination of osteoarthritis joints reveals cartilage destruction, subchondral sclerosis, osteophyte formation, and synovial inflammation [ 1, 2]. Cartilage is composed of chondrocytes and chondrocyte-produced extracellular matrix, including type II collagen (COL2A1), aggrecan, and proteoglycan 4 (Prg4, also known as lubricin) [3]. Chondrocyte senescence is an important mechanism underlying the development of osteoarthritis, resulting in reduced production of extracellular matrix proteins and an increase in the release of proinflammatory factors [ 3– 5]. Removal of senescent chondrocytes decelerates the process of osteoarthritis in surgery-induced osteoarthritis mice [6] .

Cartilage is located inside articular capsules and nourished mainly by synovial fluid [7], indicating that chondrocytes are exposed to long-term hypoxia. It is assumed that adenosine triphosphate (ATP) in chondrocytes is mainly produced by glycolysis and compensated by the tricarboxylic acid cycle in mitochondria under pathological conditions [8]. Pharmacological inhibition of glycolysis by monosodium iodoacetate accelerates the progression of osteoarthritis [9], whereas inhibition of cyclooxygenase-2 attenuates the progression of osteoarthritis [10]. It has also been reported that deleting the isoform of pyruvate kinase (PKM2), the last rate-limiting enzyme in the process of glycolysis [11], induces cell loss and reduces extracellular matrix in cultured chondrocytes under basal conditions [12] but inhibits proinflammatory cytokine interleukin-1 beta (IL-1β)-induced apoptosis [13]. Thus, the participation of glycolysis in osteoarthritis is inconclusive.

The present study was designed to investigate metabolic changes in cultured chondrocytes challenged with IL-1β. Moreover, cell apoptosis, autophagy, and reactive oxygen species were examined in cultured chondrocytes, since these are important mechanisms in the development of cartilage degeneration [ 14, 15]. It is difficult to separate the role of aging in the development of osteoarthritis since aging is a crucial player. Repeated passage of chondrocytes was used in the present study to mimic the pathological condition of osteoarthritis in the senile population. The glycolytic enzyme gene *Pkm2* was deleted in both low- and high-passage chondrocytes under basal and proinflammatory conditions.

## Materials and Methods

### Reagents

Murine IL-1β, prepared in phosphate-buffered saline (PBS; HyClone, Logan, USA) containing 0.1% bovine serum albumin (BSA; Beyotime, Shanghai, China), was purchased from R&D Systems (Minneapolis, USA). Lipopolysaccharide (LPS) and tumor necrosis factor-alpha (TNF-α) were purchased from Sigma (St Louis, USA).

### Isolation, culture, and identification of primary murine chondrocytes

Neonatal C57BL/6 mice (5 days old) were purchased from Shanghai Model Organisms Center (Shanghai, China). Immature murine articular chondrocytes (iMACs) were isolated from the knee joints of neonatal mice [16]. Briefly, after removal of the surrounding tissues, the mouse femoral condyle and tibial plateau were collected and prepared in small pieces. The cartilage fragments were digested in Dulbecco’s Modified Eagle Medium/Nutrient Mixture F-12 (DMEM/F12; Gibco, Carlsbad, USA) containing 0.2% collagenase II (Gibco) and 10% fetal bovine serum (FBS; Gibco) overnight at 37°C. After being filtered through a 70-μm strainer, chondrocytes were cultured in DMEM/F12 medium containing 10% FBS. All animal studies were approved by the Ethics Committee for Animal Research of Zhongshan Hospital (Shanghai, China).

The isolated primary chondrocytes were validated by examining the presence of sulfate proteoglycan and collagen II [16]. Cultured cells were fixed with 4% paraformaldehyde at room temperature for 15 min. To detect the presence of sulfate proteoglycan, cells were incubated in 1% Alcian blue (Servicebio, Wuhan, China) in 1 N HCl for 30 min ( Supplementary Figure S1). To examine the presence of COL2A1, the cells were permeabilized with 0.1% Triton-X-100 solution (Sigma) and blocked with 3% goat serum (Beyotime) for 1 h, followed by incubation with primary antibody against COL2A1 overnight at 4°C, and then with fluorescence-conjugated secondary antibodies for 1 h ( Supplementary Figure S1). Images were taken using an optical microscope (Olympus, Tokyo, Japan).

### Small interfering RNA transfection

When cultured cells reached 60%‒80% confluency, cells were transfected with small interfering RNAs (siRNAs) against mouse *Pkm2* and their scrambled sequences (GenePharma, Shanghai, China) which are shown in Supplementary Table S1). According to the manufacturer’s instructions, siRNA (50 nM) was delivered into cultured murine chondrocytes using Lipofectamine RNAiMAX (Invitrogen, Carlsbad, USA). The presence of PKM2 was examined 48 h after transfection. Based on the efficiency, the third siPkm2 sequence was used in the present study ( Supplementary Figure S2D–F).

### Cell culture experiments

Passage 1 chondrocytes were used in the present study, and passage 3 and 6 cells were used for the senescence study. In the present study, mRNA samples were collected 6 h after IL-1β stimulation (1 ng/mL), and protein levels, oxidative stress, and cell apoptosis were detected after 24 h of incubation.

### Real-time quantitative polymerase chain reaction (RT-qPCR)

Total RNA was extracted using Trizol (Sigma) and mRNAs were prepared with the First Strand cDNA synthesis kit (TaKaRa, Dalian, China). Real-time qPCR was performed by using Hieff qPCR SYBR Green Master Mix (Yeasen, Shanghai, China). The detailed information of primers is listed in Supplementary Table S2.

### Western blot analysis

Cell lysates were prepared using RIPA buffer (Beyotime). Protein samples were separated by 7.5%‒15% SDS-polyacrylamide electrophoresis and transferred to polyvinylidene fluoride membranes (Millipore, Billerica, USA). After being blocked in 5% nonfat milk for 1 h at room temperature, the membranes were incubated with primary antibodies ( Supplementary Table S3) overnight at 4°C, followed by incubation with corresponding secondary antibodies for 1 h at room temperature. Protein blots were visualized using a Tanon Imager 4600 system (Tanon, Shanghai, China).

### Reactive oxygen species detection

Reactive oxygen species levels were measured by cell fluorescence and fluorescence-activated cell sorting (FACS). Cultured cells were incubated with 10 μM 2′,7′-dichlorodihydrofluorescein diacetate (H2DCFDA; Sigma) at 37°C for 30 min. The fluorescent signals were detected with a fluorescence microscope (Olympus). Alternatively, 1–2×10 ^5^ cells were collected and incubated with CellROX Detection Reagent (1 μM; Thermo Fisher Scientific, Waltham, USA) for 45 min. SYTOX Dead Cell Stain solution was added to the cell suspension and incubated for 15 min. The FACS signals were examined by flow cytometry on a BD-FACSAria-III flow cytometer (BD Biosciences, Franklin Lakes, USA) and analyzed using FlowJo software (Ashland, USA).

### Cell apoptosis

Cell apoptosis was quantified using an Annexin V-FITC/PI Apoptosis Detection kit (Vazyme, Nanjing, China). A total of 1–5×10 ^5^ cells were collected using non-EDTA trypsin (Yeasen). Then, 400 μL of cell suspension was stained with 5 μL of Annexin V and 5 μL of PI for 10 min at room temperature. Apoptotic cells were identified by flow cytometry on a BD-FACSAria-III flow cytometer (BD Biosciences).

### Senescence-associated β-galactosidase (SA-β-Gal) staining assay

Cell senescence was examined using SA-β-Gal staining kit (Beyotime). Briefly, cells were fixed with 4% paraformaldehyde and incubated with SA-β-Gal staining solution at 37°C. Images were obtained using an optical microscope (Olympus).

### RNA-seq analysis

Cultured chondrocytes were transfected with siNC or siPkm2 and RNA was collected. RNA was first extracted from the cells using Trizol(Sigma). After quantification and quality control with ND-2000 (NanoDrop Technologies, Waltham, USA), 1 μg of high-quality total RNA sample was used for the transcriptome library construction by Shanghai Majorbio Biopharm Technology (Shanghai, China). Briefly, mRNA was enriched and prepared using fragmentation buffer. Reversed transcription was carried out to synthesize cDNA using random hexamer primers (Illumina, San Diego, USA) with a SuperScript double-stranded cDNA synthesis kit (Invitrogen). Then, end repair, phosphorylation and ′A′ base addition were performed to generate cDNA according to the library construction protocol from Illumina. An Illumina NovaSeq 6000 sequencer (2×150 bp read length) was used to sequence the RNA-seq sequencing library after quantification with Qubit 4.0 (Thermo Fisher Scientific). The trim and quality control were performed by fastq with default parameters. The clean reads were then separately aligned to the mouse genome using HISAT2 software. Differentially expressed genes (DEGs) were defined as |log2(fold change)|≥1 and adjusted *P*value≤0.05. The online platform of Majorbio Cloud was used for analyses. The R/Bioconductor packages pheatmap and ggplot2 were used.

### Measurement of cellular glycolytic rates by Seahorse assay

Primary chondrocyte cells (1×10 ^5^ cells/well) were seeded in the Seahorse XF cell culture microplate with DMEM/F12 medium (XF96 extracellular analyzer; Seahorse Bioscience, Wilmington, USA). Cell glycolytic rates were measured in the presence of rotenone and antimycin A (Rot/AA; 0.5 μM), as well as 2-Deoxy-D-glucose (2-DG; 50 nM), according to the manufacturer’s protocol. Wave software was used to analyze the data.

### Measurement of intracellular ATP

Intracellular ATP was measured using an ATP assay kit (Beyotime). After incubation with IL-1β (10 ng/mL) for 24 h, cell lysates were collected for ATP detection. Luminance was measured with a luminescent plate reader (Thermo Fisher Scientific). The final readout of the samples was normalized to the protein concentration and expressed as nmol/mg protein.

### Statistical analysis

All results were obtained from at least three independent experiments. Data were analyzed by GraphPad Prism 8 (San Diego, USA) and presented as the mean±SEM. Unpaired two-tailed Student’s *t* test and one-way analysis of variance (ANOVA) multiple comparisons test followed by Tukey’s test, were used for comparisons. *P* values less than 0.05 were considered statistically significant.

## Results

### PKM2 participates in regulating chondrocyte function.

In the present study, the functions of cultured chondrocytes were examined by protein expressions of MMP3, MMP13, COL2A1, and SRY-Box transcription factor (SOX)-9. MMPs are responsible for the degradation of the extracellular matrix protein COL2A1, in which MMP13 is a dominant player in the progression of osteoarthritis [17]. COL2A1 is one of the main components of the extracellular matrix [18]. SOX-9 functions during chondrocyte differentiation and is a phenotype marker for chondrocyte proliferation and differentiation [19].

**Figure 1:**
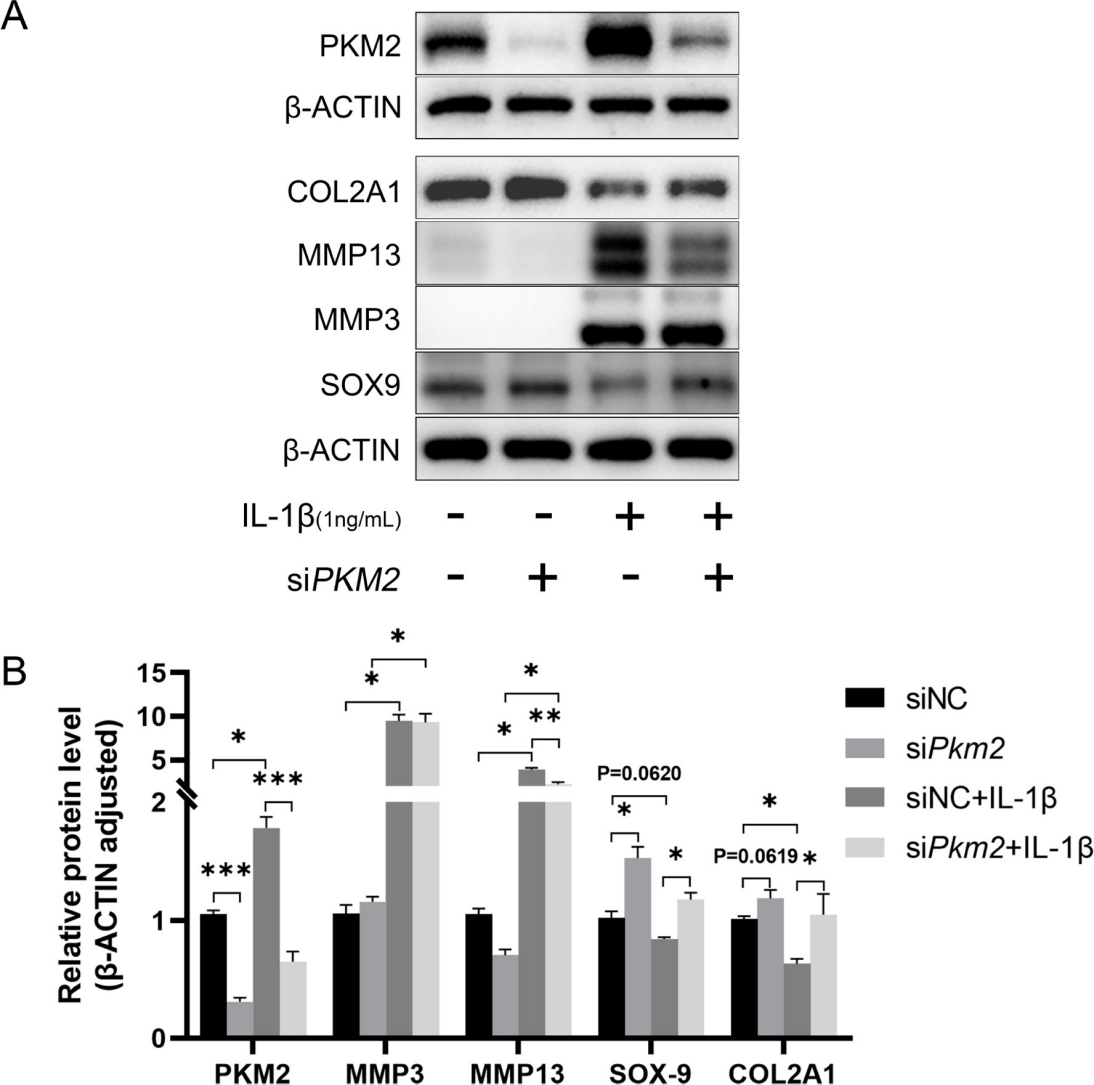
Figure 1 *Pkm2* silencing alters chondrocyte functions in cells stimulated with IL-1β Representative blots (A) and densitometric quantification (B) of PKM2, SOX-9, MMP3, MMP13, and COL2A1 proteins. n=6. *P<0.05, **P<0.01, ***P<0.001.

IL-1β stimulation significantly increased the protein expressions of MMP3 and MMP13 and downregulated COL2A1 and SOX-9 protein expressions ( Figure 1A,B). Silencing of *Pkm2* reduced the upregulation of MMP13 but not MMP3 in cells challenged with IL-1β. Silencing of *Pkm2* significantly increased COL2A1 and SOX-9 protein expressions under basal and stimulated conditions ( Figure 1A,B).

### PKM2 does not participate in IL-1β-induced oxidative stress, autophagy, or apoptosis

**Figure 2:**
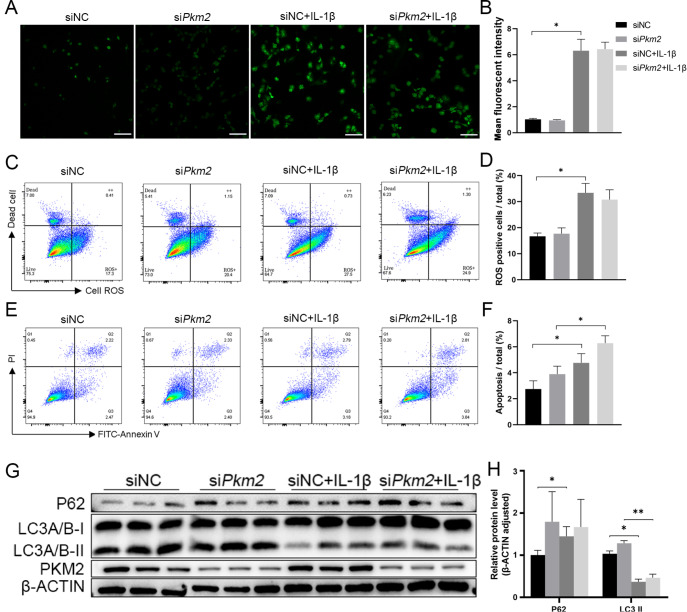
Figure 2 *Pkm2* silencing does not alter ROS levels, cell apoptosis, or autophagy (A) Measurement of reactive oxidative stress, a reduced form of H2DCFDA, in cultured cells stimulated with IL-1β and (B) quantification of mean fluorescence intensity, n=6. (C) Measurement of CellROX/SYTOX signals by FACS in cultured cells stimulated with IL-1β and (D) quantification of ROS-positive cells, n=3. (E) Annexin V/PI signals examined by FACS and (F) quantification of apoptotic cells, n=6. Representative blots (G) and densitometric quantification (H) of LC3A/B and p62 proteins, n=3. *P<0.05, **P<0.01. Scale bar=500 μm.

IL-1β stimulation significantly increased oxidative stress in chondrocytes, as measured by cell fluorescence and FACS. Silencing of *Pkm2* did not reduce IL-1β-induced oxidative stress ( Figure 2A–D). Stimulation with IL-1β significantly induced cell apoptosis, as shown by increased Annexin V signals. Silencing of *Pkm2* further increased apoptotic signals in cells stimulated with IL-1β ( Figure 2E,F).

Stimulation with IL-1β significantly increased sequestosome-1 (p62) protein expression and decreased microtubule-associated protein 1A/1B light chain 3 (LC3A/B) protein expression, two key players in the process of autophagy. Silencing of *Pkm2* did not affect the protein expressions of p62 and LC3A/B in cells stimulated with IL-1β ( Figure 2G,H).

Since silencing of *Pkm2* did not play a role in inflammation, it is assumed that the protective role of *Pkm2* silencing in chondrocytes occurs under quiescent conditions.

### Silencing of *Pkm2* affects senescent p16 ^INK4a^ protein in chondrocytes

**Figure 3:**
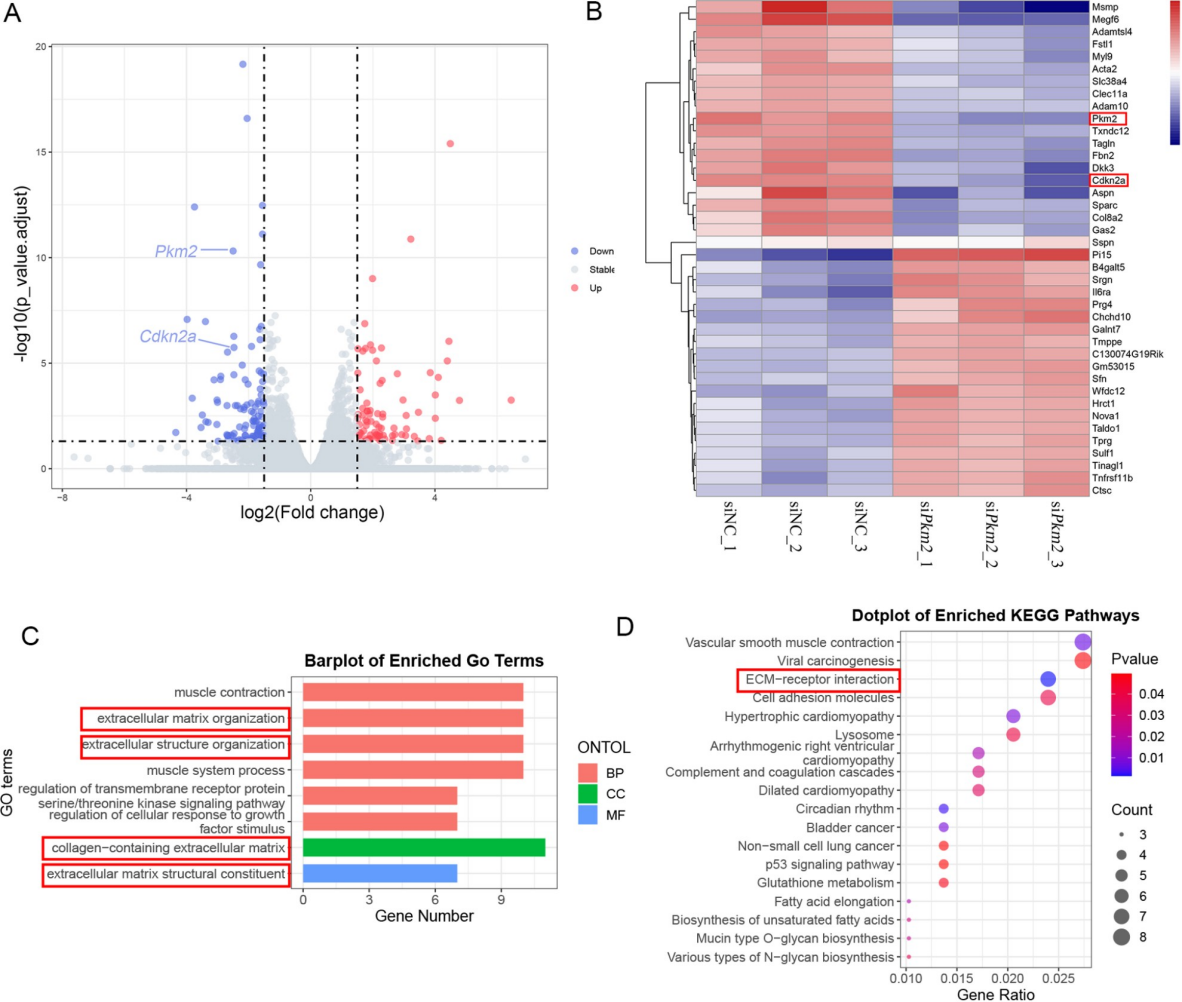
Figure 3 Bioinformatics analysis of DEGs in primary chondrocytes with *Pkm2* silencin (A) Volcano plot of DEGs. (B) Heatmap showing the top 20 upregulated or downregulated DEGs. (C) GO terms enriched for DEGs. (D) KEGG pathway analysis for DEGs.

To further understand the role of PKM2 in chondrocytes, RNA sequencing was conducted in primary chondrocytes with *Pkm2* silencing. There were 95 differentially expressed genes (DEGs), 40 upregulated and 55 downregulated ( Figure 3A,B). Modules of extracellular matrix organization, extracellular structure organization, collagen-containing extracellular matrix, and extracellular matrix structural constituent were enriched ( Figure 3C). The ECM-receptor interaction pathway was markedly enriched in the KEGG pathway analysis ( Figure 3D).

RNA sequencing analysis revealed that p16 ^INK4a^, known as Cdkn2a, was downregulated in cells transfected with siPkm2 compared with that in siNC-transfected chondrocytes ( Figure 3 A,B).

RT-qPCR confirmed the increased mRNA expressions of *Prg4* and extracellular heparin sulfate 6-O ( *Sulf1*) and reduced mRNA expression of a disintegrin and metalloproteinase 10 ( *Adam10*) and follistatin-like protein 1 ( *Fstl1*) in quiescent and stimulated chondrocytes with *Pkm2* silencing ( Supplementary Figure S3A‒D).

### PKM2 regulates chondrocyte senescence

**Figure 4:**
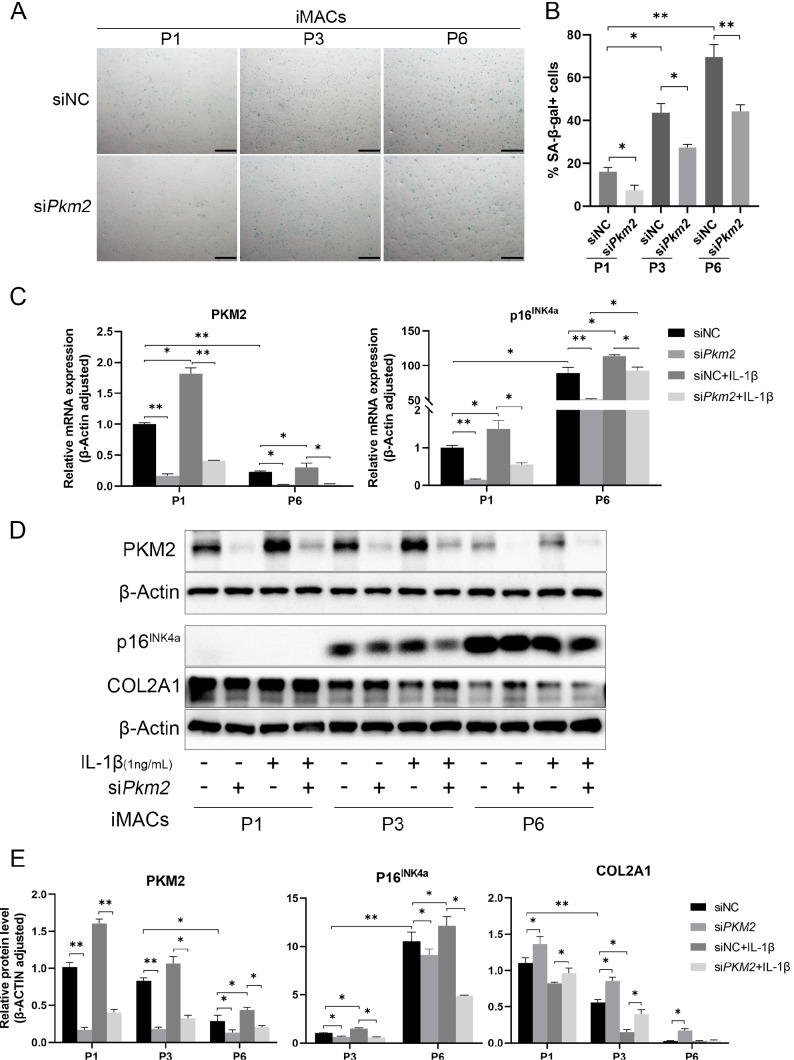
Figure 4 *Pkm2* silencing decelerates chondrocyte senescence (A) SA-β-Gal activity and (B) quantification of SA-β-Gal-positive chondrocytes in cultured cells, n=6. (C) mRNA expressions of p16INK4a and PKM2 in low- and high-passage chondrocytes stimulated with IL-1β, n=3. Representative blots (D) and densitometric quantification (E) of PKM2, p16INK4a, and COL2A1 proteins in low- and high-passage chondrocytes stimulated with IL-1β, n=3. *P<0.05, **P<0.01. Scale bar=50 μm.

Protein p16 ^INK4a^ is a biomarker of cellular aging [ 3, 4, 20]. High-passage chondrocytes had higher SA-β-Gal activity than low-passage chondrocytes ( Figure 4A,B), which was reduced by *Pkm2* silencing ( Figure 4A,B).

Both mRNA and protein expressions of p16 ^INK4a^ were upregulated in chondrocytes in a passage-dependent manner ( Figure 4C–E). In high-passage cells, IL-1β stimulated PKM2 and p16 ^INK4a^ upregulation, which were in the same pattern as those in low-passage chondrocytes. Silencing of *PKM2* reduced p16 ^INK4a^ expression under quiescent and stimulated conditions ( Figure 4C–E).

Under quiescent conditions, high-passage chondrocytes had reduced protein expression of COL2A1. In line with the results of low-passage cells, silencing of *Pkm2* increased COL2A1 protein expression under quiescent but not stimulated conditions in high-passage chondrocytes ( Figure 4D–E).

### PKM2 is important in cell senescence but not in IL-1β-induced inflammation in chondrocytes

PKM2, a rate-limiting enzyme in glycolysis, is important for ATP generation [ 21, 22]. In the present study, silencing of *Pkm2* reduced the mRNA levels of fructose-bisphosphate aldolase c ( *Aldoc*), glucose-6-phosphate isomerase 1 ( *Gpi1*), and phosphoglycerate kinase 1 ( *Pgk1*), but not lactate dehydrogenase A ( *Ldha*), phosphofructokinase muscle isoform ( *Pfkm*), or hexokinase 2 ( *Hk2*), in quiescent cells, both in low- and high-passage cells ( Supplementary Figure S4A–F). Stimulation with IL-1β significantly increased the mRNA expressions of these glycolytic enzymes, including Aldoc, Ldha, Gpi1, Hk2, and Pgk1, which were not affected by *Pkm2* silencing ( Supplementary Figure S4A–F).

The mRNA expression levels of genes in the tricarboxylic acid cycle were examined. Silencing of *Pkm2* increased the mRNA levels of citrate synthase ( *Cs*), dihydrolipoamide s-succinyltransferase ( *Dlst*), isocitrate dehydrogenase 2 ( *Idh2*), *Idh3a*, *Idh3b*, oxoglutarate dehydrogenase ( *Ogdh*), and mitochondrial transcription factor A ( *Tfam*) in low-passage cells but not in high-passage cells ( Supplementary Figure S4F‒P).

Stimulation with IL-1β significantly increased the mRNA expression of *Dlst* but not other players in the tricarboxylic acid cycle in low-passage cells ( Supplementary Figure S4F‒P). Stimulation with IL-1β did not change the mRNA expression of the above mentioned players in the tricarboxylic acid cycle in high-passage cells ( Supplementary Figure S4F‒P).

**Figure 5:**
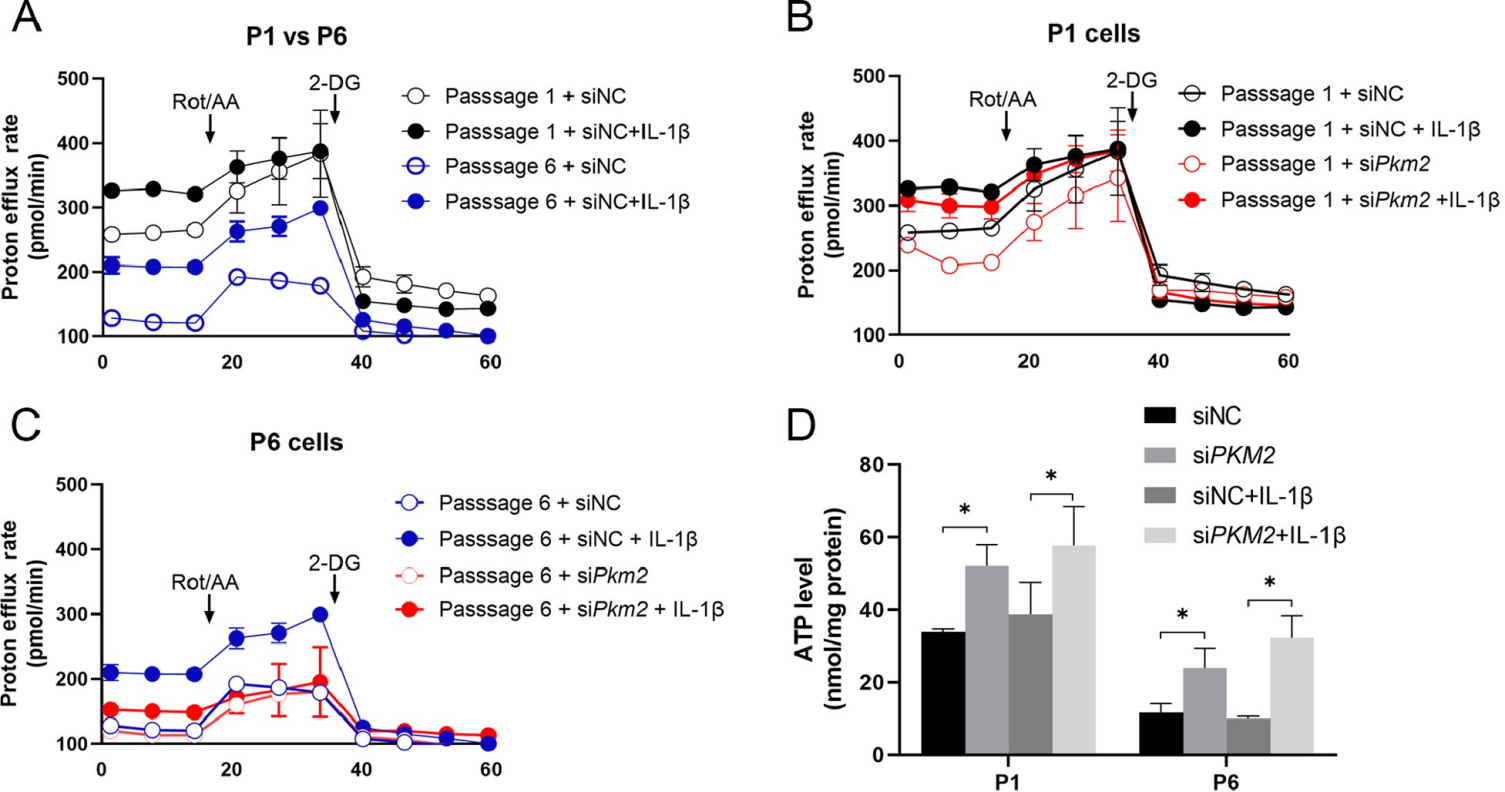
Figure 5 Glycolytic rate measured by Seahorse assay in low- and high-passage chondrocytes stimulated with IL-1β (A) Glycolytic rate measured by Seahorse assay in low- and high-passage chondrocytes stimulated with IL-1β. (B,C) Glycolytic rates in low-passage and high-passage cells stimulated with IL-1β, n=4. (D) ATP levels in low- and high-passage cells stimulated with IL-1β, n=6. *P<0.05.

To further confirm the protective role of *Pkm2* in chondrocytes, the glycolytic rates of cells under both basal and stimulated conditions were measured by Seahorse assay. Compared with low-passage cells, high-passage cells had reduced proton efflux rates, an indicator of the extracellular level of lactate ( Figure 5A). IL-1β stimulation significantly increased proton efflux rates in low- and high-passage chondrocytes ( Figure 5A).

The proton efflux rates in low-passage cells were comparable when the mitochondrial respiratory chain was blocked, comparing quiescent cells to the stimulated cells ( Figure 5B). In high-passage chondrocytes, IL-1β stimulated a higher proton efflux rate when the mitochondrial respiratory chain was blocked ( Figure 5B).

Silencing of *Pkm2* reduced proton efflux rates in low-passage cells, which were not significantly affected when the mitochondrial respiratory chain was blocked ( Figure 5C). Silencing of *Pkm2* reduced proton efflux rates in high-passage cells under both quiescent and stimulated conditions when the mitochondrial respiratory chain was blocked ( Figure 5A,B).

The basal levels of ATP were increased in both low- and high-passage chondrocytes with *PKM2* silencing. IL-1β stimulation did not significantly increase ATP production in either low- or high-passage cells ( Figure 5D).

## Discussion

In the present study, we revealed that glycolysis is the primary energy-producing setting of chondrocytes under quiescent conditions and shifts to the tricarboxylic acid cycle when cells are silenced with *Pkm2*. In the process of cell senescence, the glycolytic enzyme PKM2 is a key player, but not in the process of inflammation. The protective role of PKM2 in senescence is attributed to the downregulation of p16 ^INK4a^ protein.

MMPs and ADAMs degrade extracellular matrix proteins, including fibrillar collagen and aggrecan, respectively. In addition, the expressions of Prg4, Sulf1, Adam10, and Fstl1 were also examined in quiescent and stimulated chondrocytes. Prg4, an important component of extracellular matrix proteins, is synthesized and secreted by chondrocytes [23]. Prg4 protein modulates cartilage homeostasis by reducing friction between articular cartilage surfaces [24], since deleting Prg4 induces chondrocyte apoptosis [ 24, 25]. Sulf1 hydrolyses sulfates from heparan sulfate proteoglycans and regulates the contents of the extracellular matrix [ 26, 27]. Mice lacking the Sulf1 protein exhibit spontaneous cartilage degeneration and osteoarthritis [28]. The Adam10 protein, which degrades aggrecan, is increased in degenerated cartilage and osteoarthritis cartilage [ 29, 30]. Enhanced expression of Fstl1 suppresses chondrogenesis, resulting in cartilage destruction [ 31, 32]. In the present study, chondrocytes stimulated with IL-1β exhibited downregulation of COL2A1 and upregulation of MMP13, Prg4, Sulf1, Adam10, and Fstl1, supporting that enhanced inflammation is a critical mechanism underlying the progression of osteoarthritis [33].

Stimulation with IL-1β increased oxidative stress, enhanced autophagy, and induced cell apoptosis, confirming that these biological processes are involved in the development of osteoarthritis [ 14, 15, 34]. However, silencing of *Pkm2* did not change IL-1β-induced oxidative stress, autophagy, or cell apoptosis. Silencing of *Pkm2* exerted similar patterns of chondrocyte function proteins under both basal and stimulated conditions. Thus, these data imply that glycolysis is the dominant player under basal conditions.

The tricarboxylic acid cycle occurs in mitochondria and is the primary energy source for cell migration, proliferation, and function [35]. Nevertheless, when cells are exposed to hypoxia, glycolysis is more responsible for energy production [36]. Since the cartilage inside the articular capsules is exposed to long-term hypoxia, glycolysis is assumed to be the energy-producing setting of chondrocytes [37]. In the present study, increased level of ATP, together with the upregulation of genes in the tricarboxylic acid cycle in cells silenced with *Pkm2*, suggested that the tricarboxylic acid cycle in mitochondria compensates for the impairment of glycolysis.

It is acknowledged that laboratory-induced aging does not fully reproduce the pathophysiological changes in the course of nature. However, it is difficult to separate the role of aging in the process of osteoarthritis, since aging is an important underlying mechanism of the disease. In the laboratory, cell aging is induced by chronic oxidative stress and repeated passage, two common approaches. Chondrocytes are terminally differentiated cells, and their differentiated phenotypes are lost during senescence [16]. Indeed, primary chondrocytes rapidly lose their differentiated phenotype upon repeated passages, showing reduced expressions of COL2A1 and degrading proteins. Importantly, increased SA-β-Gal activity and enhanced p16 ^INK4a^ in high-passage chondrocytes were observed in the present study, supporting that chondrocyte senescence was successfully induced.

In addition to increased SA-β-Gal activity and enhanced p16 ^INK4a^ expression, in high-passage chondrocytes, deletion of PKM2 reduced p16 ^INK4a^ protein expression, decelerated chondrocyte senescence, and partially restored chondrocyte functions, as shown by decreased SA-β-Gal activity and increased expression of COL2A1 in cultured cells, indicating that PKM2 protein is beneficial for chondrocyte senescence.

To mimic the pathological conditions of osteoarthritis in the aged population, stimulation with IL-1β was also applied in high-passage chondrocytes. Consistent with the observation in low-passage cells, silencing of *Pkm2* had similar patterns on protein changes of p16 ^INK4a^ under both basal and stimulated conditions, supporting the note that PKM2 does not participate in inflammation.

Notably, increased expression of p16 ^INK4a^ is also observed in quiescent cells stimulated with IL-1β, suggesting that the upregulation of p16 ^INK4a^ by proinflammatory cytokines or repeated passages occurs through different mechanisms, including the NF-κB signaling pathway [ 36, 38– 40] .

In summary, metabolic changes respond quickly to physiological and pathological stimulations. Glycolysis is vital in quiescent chondrocytes, and the tricarboxylic acid cycle steps forward when the protective effects of glycolysis recede under pathological conditions. *Pkm2* silencing decelerates chondrocyte senescence by regulating p16 ^INK4a^. Therefore, targeting the *Pkm2* gene is a promising clinical therapy and sheds light on the treatment of age-related osteoarthritis.

## Supporting information

22677supplementary_file_revision

## Supplementary Data

Supplementary data is available at *Acta Biochimica et Biophysica Sinica* online.
