# Supplementary material for: Glycolytic enzyme PKM2 regulates cell senescence but not inflammation in the process of osteoarthritis: PKM2 regulates chondrocyte senescence via p16 INK4a transcription
Source: Acta Biochim Biophys Sin (Shanghai). 2023 Jul 31;55(9):1425–33. doi: 10.3724/abbs.2023062 (PMC10520488; doi:10.3724/abbs.2023062)
Supplement: 22677supplementary_file_revision [file 22677supplementary_file_revision.pdf]

Supplementary Table S1. siRNA information

Target gene	Sense sequence (5′→3′)	Antisense sequence (5′→3′)
Scramble	UUCUCCGAACGUGUCACGUTT	ACGUGACACGUUCGGAGAATT
si*Pkm2* #1	CUGGCAUCAUUUGUACCAUTT	AUGGUACAAAUGAUGCCAGTT
si*Pkm2* #2	CAGAGACCAUCAAGAAUGUTT	ACAUUCUUGAUGGUCUCUGTT
si*Pkm2* #3	CAUGCUGUCUGGAGAAACATT	UGUUUCUCCAGACAGCAUGTT

Supplementary Table S2. The sequences of primers (mouse) for PCR analysis

Gene	Primer sequence (5′→3′)	
Pkm2	Sense	TTGCAGCTATTCGAGGAACTCCG
	Anti-sense	CACGATAATGGCCCCACTGC
Cdkn2a	Sense	GTACCCCGATTCAGGTGAT
	Anti-sense	TTGAGCAGAAGAGCTGCTACGT
β-Actin	Sense	GGCTGTATTCCCCTCCATCG
	Anti-sense	CCAGTTGGTAACAATGCCATGT
Gpi1	Sense	CTCAAGCTGCGCGAACTTTTT
	Anti-sense	GGTTCTTGGAGTAGTCCACCAG
Ldha	Sense	CAAAGACTACTGTGTAACTGCGA
	Anti-sense	TGGACTGTACTTGACAATGTTGG
Aldoc	Sense	AGAAGGAGTTGTCGGATATTGCT
	Anti-sense	TTCTCCACCCCAATTTGGCTC

Pgk1	Sense	ATGTCGCTTTCCAACAAGCTG
	Anti-sense	GCTCCATTGTCCAAGCAGAAT
Hk2	Sense	TGATCGCCTGCTTATTCACGG
	Anti-sense	AACCGCCTAGAAATCTCCAGA
Pfkm	Sense	TGTGGTCCGAGTTGGTATCTT
	Anti-sense	GCACTTCCAATCACTGTGCC
Tfam	Sense	AACACCCAGATGCAAAACTTTCA
	Anti-sense	GACTTGGAGTTAGCTGCTCTTT
Sulf1	Sense	TTTGTCGCAACGGCATCAAG
	Anti-sense	GGAGGCATTGGGGTACAGTT
Adam10	Sense	ATGGTGTTGCCGACAGTGTTA
	Anti-sense	GTTTGGCACGCTGGTGTTTTT
Fstl1	Sense	AATGGCAAGACCTACCTCAACC
	Anti-sense	GTGCCCATCATAATCAACCTGG
Prg4	Sense	TTTTGGCCGGGAGACTCAATC
	Anti-sense	CAGCGTAGTCAGTCCATCCAC
Cs	Anti-sense	GGACAATTTTCCAACCAATCTGC
	Sense	TCGGTTCATTCCCTCTGCATA
Idh1	Anti-sense	ATGCAAGGAGATGAAATGACACG
	Sense	GCATCACGATTCTCTATGCCTAA
Idh2	Anti-sense	GGAGAAGCCGGTAGTGGAGAT
	Sense	GGTCTGGTCACGGTTTGGAA
Idh3a	Anti-sense	TGGGTGTCCAAGGTCTCTC
	Sense	TCCCACTGAATAGGTGCTTTG
Idh3b	Anti-sense	AGGCACAAGATGTGAGGGTG
	Sense	CAGCAGCCTTGAACACTTCC

Idh3g	Anti-sense	GGTGCTGCAAAGGCAATGC
	Sense	TATGCCGCCCACCATACTTAG
Ogdh	Anti-sense	GTTTCTTCAAACGTGGGGTTCT
	Sense	GCATGATTCCAGGGGTCTCAAA
Dlst	Anti-sense	GGAACTGCCCTCTAGGGAGA
	Sense	GACGCTACCACTGTTAATGACC
Dld	Anti-sense	GAGCTGGAGTCGTGTGTACC
	Sense	CCTATCACTGTCACGTCAGCC

Supplementary Table S3. Antibody information used in this study

Antibody	Company	Catalog	Application (dilution)
Collagen II	Proteintech	28459-1-AP	WB (1:500)/IF (1:100)
MMP13	Proteintech	18165-1-AP	WB (1:1000)
MMP3	Abcam	EP1186Y	WB (1:1000)
SOX-9	Proteintech	67439-1-Ig	WB (1:1000)
LC3A/B	Cell Signaling Technology	D3U4C	WB (1:1000)
p62	Cell Signaling Technology	5114	WB (1:1000)
β-ACTIN	Abcam	ab8226	WB (1:1000)
PKM2	Cell Signaling Technology	D78A4	WB (1:10000)
$p16^{INK4a}$	Cell Signaling Technology	E5F3Y	WB (1:500)
AffiniPure Goat Anti-Mouse IgG (H+L)	Jackson ImmunoResearch	115-005-003	WB (1:10000)
AffiniPure Goat Anti-Rabbit IgG (H+L)	Jackson ImmunoResearch	111-005-003	WB (1:10000) /IF (1:800)

Supplementary figure legends

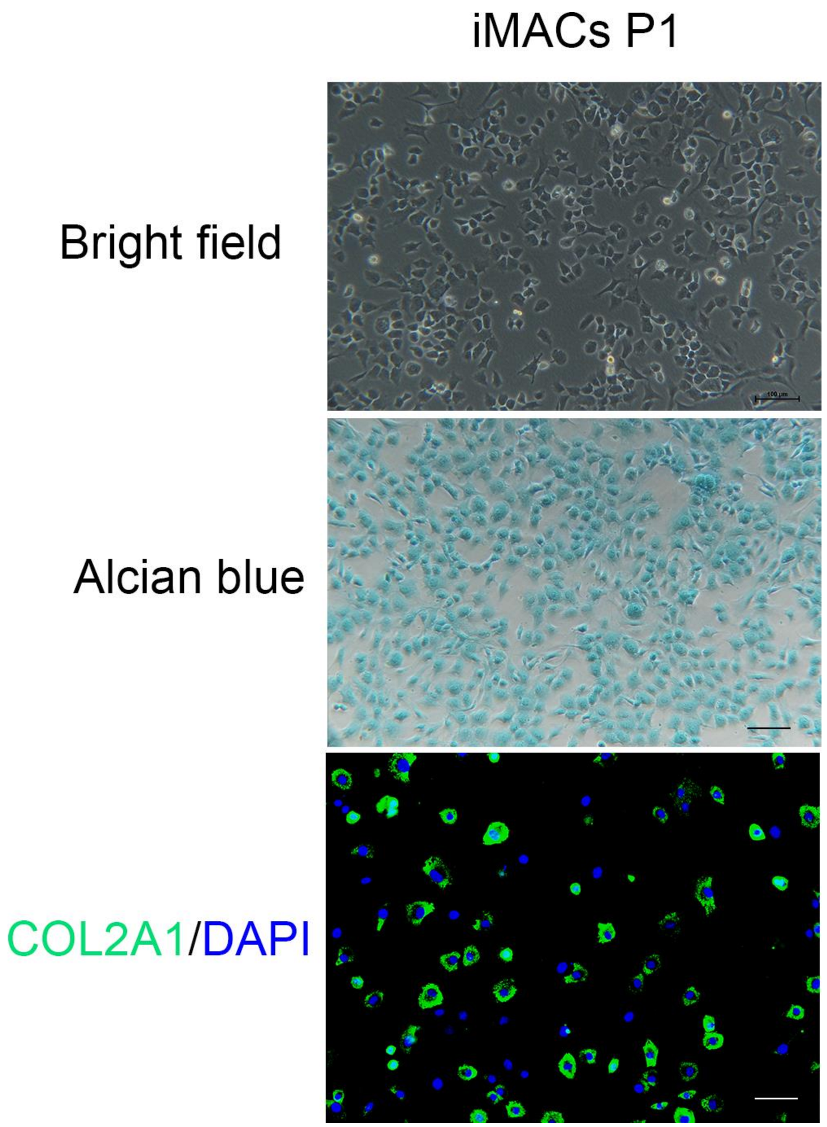

Supplementary Figure S1. Identification of iMACs via morphology (top), Alcian blue (middle) and immunofluorescence of COL2A1 (bottom) COL2A1 labeled green, and DAPI labeled blue. Scale bar:100 μm.

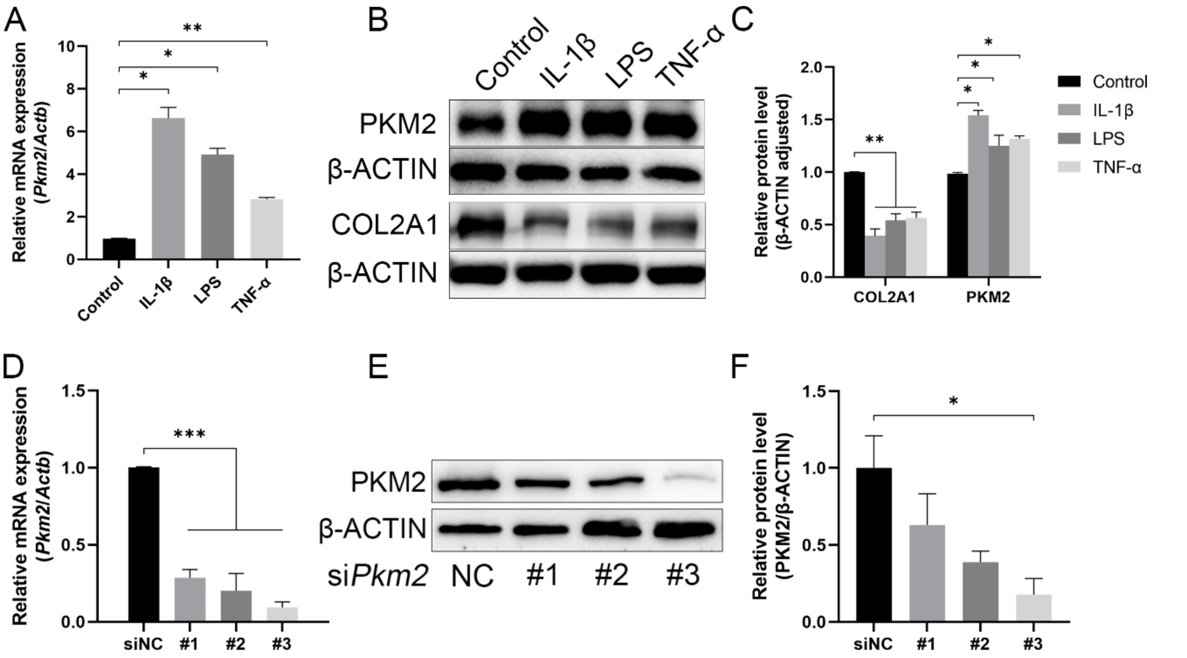

Supplementary Figure S2. PKM2 expression is upregulated by pro-inflammatory factors in chondrocytes (A) mRNA expression of *PKM2* in chondrocytes stimulated with IL-1β (1 ng/mL), LPS (10 μg/mL), and TNF-α (10 ng/mL) for 24 h. *n*=6. Representative blots (B) and densitometric quantification (C) of PKM2 and COL2A1 proteins in chondrocytes stimulated with IL-1β, LPS, and TNF-α, respectively. *n*=3. (D) mRNA expression of PKM2 in chondrocytes transfected with siPkm2 for two days. Representative blots (E) and densitometric quantification (F) of PKM2 protein in chondrocytes under basal condition. *n*=6. **P*< 0.05, ***P*< 0.01.

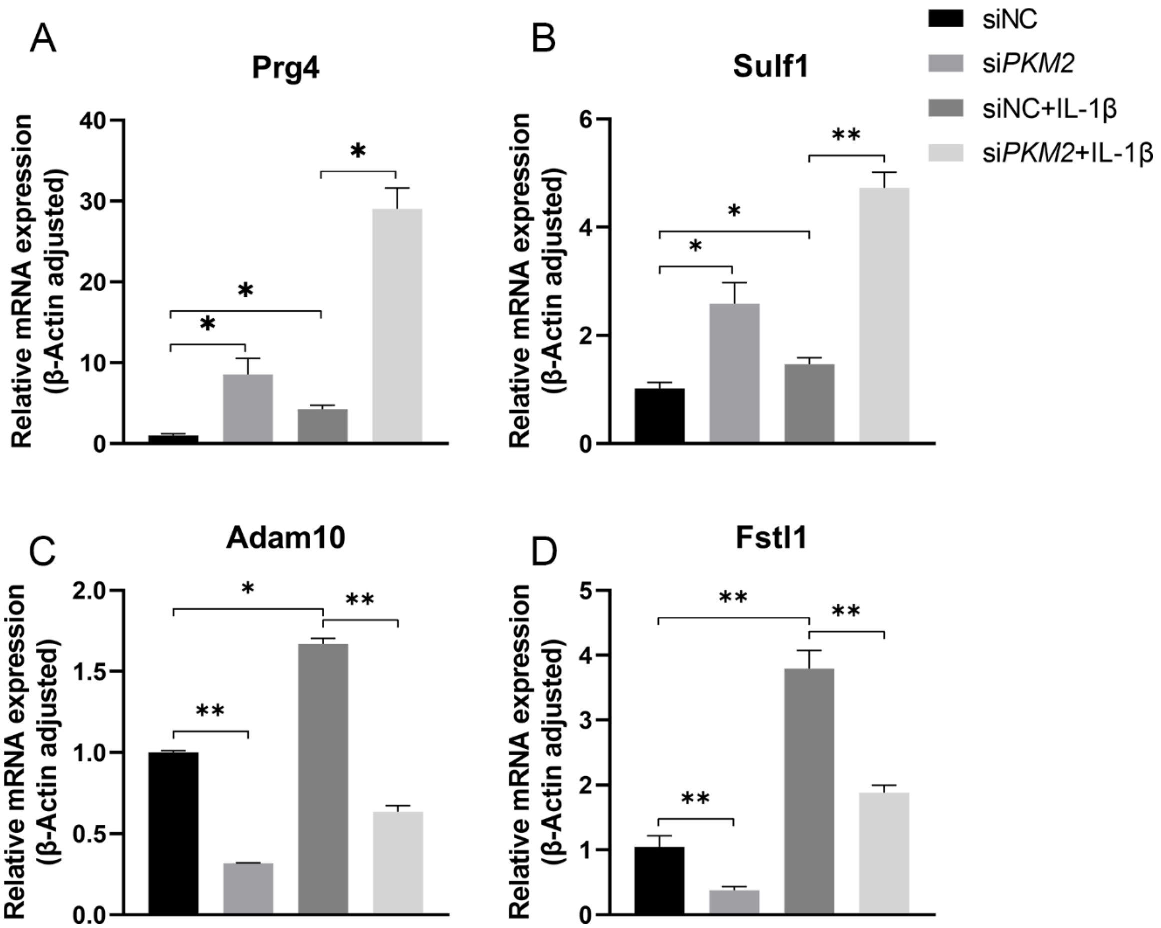

Supplementary Figure S3. mRNA expressions of (A) *Prg4*, (B) *Sulf1*, *Adam10*, and *Fstl1* in cultured chondrocytes simulated with IL-1β (1 ng/mL) for 6 h *n*=6. **P*< 0.05, ***P*< 0.01.

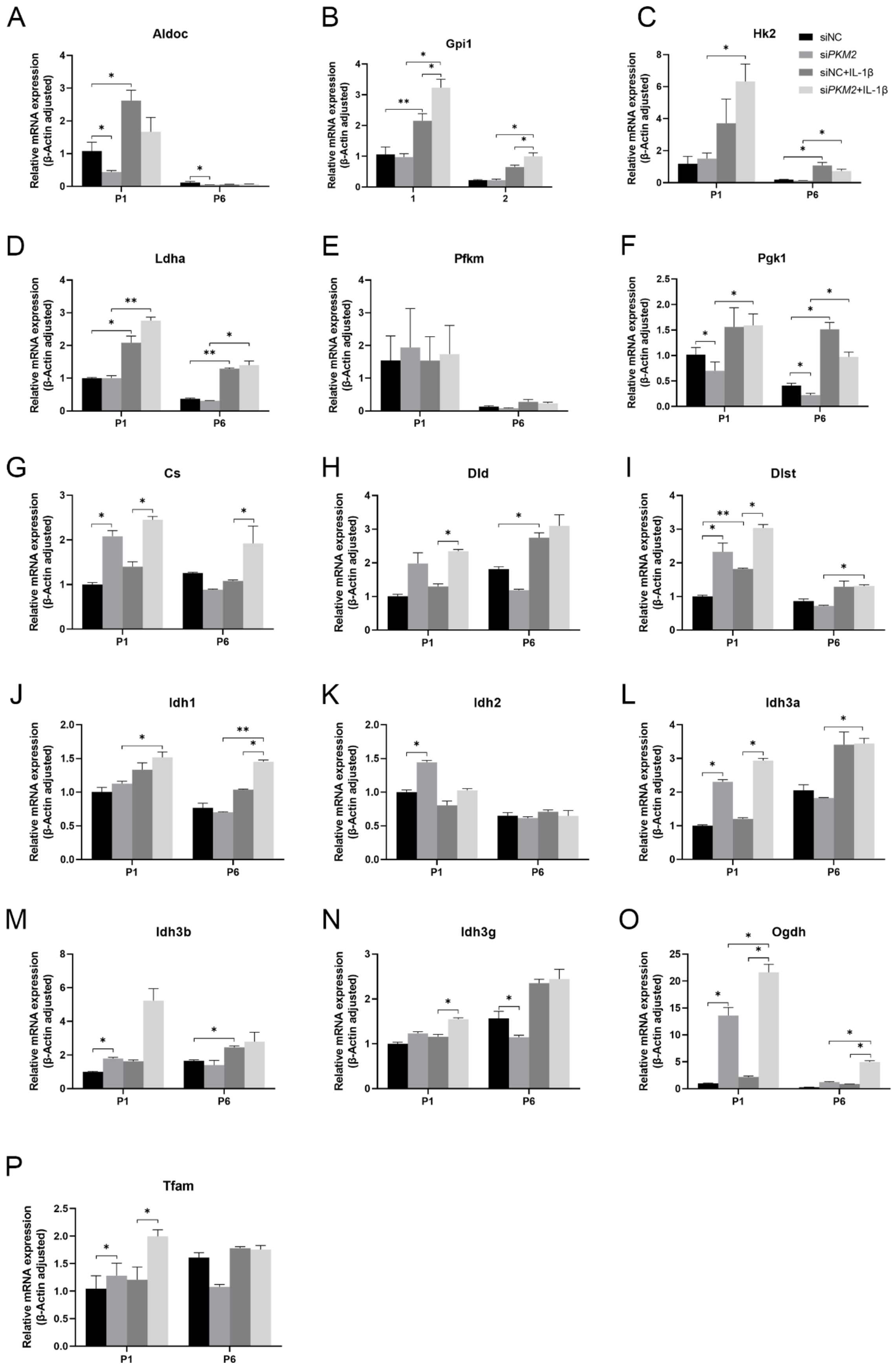
A
Aldoc
B
Gpi1
C
Hk2
siNC
siPKM2
siNC+IL-1β
siPKM2+IL-1β
D
Ldha
E
Pfkm
F
Pgk1
G
Cs
H
Dld
I
Dlst
J
Idh1
K
Idh2
L
Idh3a
M
Idh3b
N
Idh3g
O
Ogdh
P
Tfam
Relative mRNA expression (β-Actin adjusted)
P1
P6

Supplementary Figure S4. mRNA expressions of glycolytic proteins and mitochondrial tricarboxylic acid cycle-related proteins in cultured chondrocytes stimulated with IL-1β (1 ng/mL) for 6 h Quantitative PCR analysis of (A) *Aldoc*, (B) *Gpi1*, (C) *Hk2*, (D) *Ldha*, (E) *Pfkm*, and (F) *Pgk1*, (G) *Cs*, (H) *Dld*, (I) *Dlst*, (J) *Idh1*, (K) *Idh2*, (L) *Idh3a*, (M) *Idh3b*, (N) *Idh3g*, (O) *Ogdh*, and (P) *Tfam* in chondrocytes. n=6. *P< 0.05, **P<0.01.
